# Cystathionine β-synthase deficiency: different changes in proteomes of thrombosis-resistant *Cbs*^−/−^ mice and thrombosis-prone *CBS*^−/−^ humans

**DOI:** 10.1038/s41598-020-67672-5

**Published:** 2020-07-01

**Authors:** Marta Sikora, Izabela Lewandowska, Łukasz Marczak, Ewa Bretes, Hieronim Jakubowski

**Affiliations:** 1 European Centre for Bioinformatics and Genomics, Institute of Bioorganic Chemistry, 61-704 Poznan, Poland; 2 Department of Biochemistry and Biotechnology, University of Life Sciences, 60-632 Poznan, Poland; 3 Department of Biochemistry, Microbiology and Molecular Genetics, Rutgers-New Jersey Medical School, International Center for Public Health, 225 Warren Street, Newark, NJ 07103 USA

**Keywords:** Genetics, Molecular biology, Molecular medicine

## Abstract

Cystathionine β-synthase (CBS)-deficient patients are prone to vascular thrombosis. In contrast, *Cbs*^−/−^ mice show no abnormalities in blood coagulation. To identify molecular basis underlying these disparately different thrombotic phenotypes, we analyzed plasma proteomes of *Cbs*^−/−^ vs. *Cbs*^+/+^ mice (8-month-old, 12/group, sex-matched) and *CBS*^−/−^ vs. *CBS*^+/+^ humans (37 ± 7-year-old, 10–14/group, sex-matched) using label-free mass spectrometry. We identified 117 and 41 differentiating plasma proteins in *Cbs*^−/−^ mice and *CBS*^−/−^ humans, respectively. Twenty-one proteins were shared between *CBS*^−/−^ humans and *Cbs*^−/−^ mice, with sixteen changed in the opposite direction. Proteins involved in blood coagulation and complement/coagulation cascades represented a greater fraction of the differentiating proteins in *CBS*^−/−^ patients (51%) than in *Cbs*^−/−^ mice (21%). Top canonical pathways, identified by Ingenuity Pathways Analysis, such as LXR/RXR, FXR/RXR activation (− log[*P*-value] = 30–31) and atherosclerosis signaling (− log[*P*-value] = 10–11) were similarly affected in *Cbs*^−/−^ mice and *CBS*^−/−^ humans. The Coagulation System was affected stronger in *CBS*^−/−^ humans than in *Cbs*^−/−^ mice (− log[*P*-value] = 15 vs. 10, respectively) while acute phase response and complement system were affected stronger in *Cbs*^−/−^ mice (− log[*P*-value] = 33 and 22, respectively) than in humans (− log[*P*-value] = 22 and 6, respectively). Other pathways, including IL-7 signaling and B cell development were affected only in *Cbs*^−/−^ mice. Taken together, our findings suggest that differences in these processes, in particular in the Coagulation System, could account for the thrombotic phenotype in *CBS*^−/−^ patients and the absence of thrombosis in *Cbs*^−/−^ mice. Overall, our findings suggest that *Cbs*^−/−^ mice have a better adaptive response to protect from prothrombotic effects of hyperhomocysteinemia than *CBS*^−/−^ humans.

## Introduction

Patients with cystathionine β-synthase (CBS) deficiency, a rare inborn error of metabolism caused by mutations in the *CBS* gene, have severely elevated levels of the sulfur-amino acid homocysteine (Hcy)^1^ and its metabolites^2^. The only known source of Hcy in the human body is the dietary protein methionine, which is converted to Hcy in a sequence of three consecutive reactions with AdoMet and AdoHcy as intermediates. Hcy is further metabolized via three pathways, affording cysteine, methionine, or Hcy-thiolactone^3^. The metabolic conversion of Hcy to cysteine is impaired in CBS-deficient patients, causing severe hyperhomocysteinemia (HHcy)^1^ and enhancing the synthesis of Hcy-thiolactone^2^. The dysregulation of Hcy metabolism in human CBS deficiency is accompanied by pathological changes in many organs, including the cardiovascular system. *CBS*^−/−^ patients have a blood clotting defect^1^ underlying their high susceptibility to vascular thromboembolism (50% chance by the age of 30), which is the major cause of morbidity and mortality^1^. Although the mechanisms leading to the onset of thromboembolism in *CBS*^−/−^ patients are not fully understood^4^, 32% of those patients suffer thromboembolic stroke and 4% have myocardial infarction^1^. Other vascular thromboembolic incidents occur in the peripheral veins (51%) and arteries (11%) of *CBS*^−/−^ patients^1^.

To study mechanisms underlying the pathology associated with elevated Hcy, the *Cbs* gene has been deleted in mice. The *Cbs*^−/−^ homozygous mutants showed a neonatal phenotype due to liver dysfunction and only a few percent of them survived to adulthood^5^. The neonatal lethality was circumvented by providing betaine in the drinking water^6^ or by creation of transgenic *Cbs*^−/−^ mice harboring wild type or mutant versions of the human *CBS* gene under control of the zinc-inducible metallothionein promoter, *Tg-I278T Cbs*^−/−^ mouse^7^. The *Cbs*^−/−^ mice recapitulate most, but not all, of the pathological changes observed in human *CBS*^−/−^ patients^6,7^ including the reduced life span^7^.

Surprisingly, *Tg-I278T Cbs*^−/−^ mice that have severe HHcy (plasma total Hcy 250 μM) do not have the thrombotic phenotype^7,8^ found in *CBS*^−/−^ patients^1,4,9,10^. This was established by finding no differences between *Tg-I278T Cbs*^−/−^ vs. *Tg-I278T Cbs*^+/+^ mice in the prothrombin time, activated partial thromboplastin time^7^, bleeding time, and the occlusion time in the chemical FeCl_3_ vascular injury model^8^ that was used to demonstrate impaired fibrinolysis caused by the formation of *S*-Hcy-annexin 2 in wild type mice with plasma total Hcy elevated (to 70 μM) by dietary intervention^11^. *Tg-I278T Cbs*^−/−^ mice also do not show elevated arterial or venous thrombosis in other assays, including photochemical injury of the carotid artery, injury of the carotid artery and mesenteric arterioles, and ligation of the inferior vena cava^8^. Hemostatic and hemodynamic parameters are also not affected in *Tg-I278T Cbs*^−/−^ mice.

Different susceptibilities of *CBS*^−/−^ patients and *Cbs*^−/−^ mice to thrombosis might be reflected in their proteomes. Previous work has identified CBS deficiency-responsive proteins in human plasma^12^. In the present work we have used label-free mass spectrometry and the Ingenuity Pathway Analysis resources to interrogate plasma proteomes of thrombosis-resistant *Cbs*^−/−^ mice and compared them with plasma proteomes of thrombosis-prone *CBS*^−/−^ humans.

## Results

Total Hcy levels in *CBS*^−/−^ patients and *Cbs*^−/−^ mice were severely elevated compared to corresponding unaffected controls (71.2 ± 55.5 vs. 9.7 ± 5.9 μM in human plasma^12^; 236 ± 80 vs. 10.2 ± 5.3 μM in mouse plasma), as expected^13,14^. In each group of subjects, label-free nanoLC-MS/MS mass spectrometry identified 196–198 proteins in human and mouse plasma with a minimum of two peptides and 1% false discovery rate (FDR). The overlap between duplicate injections was > 90% at the protein level as determined by the Proteome Discoverer (PD) analysis.

**Figure 1:**
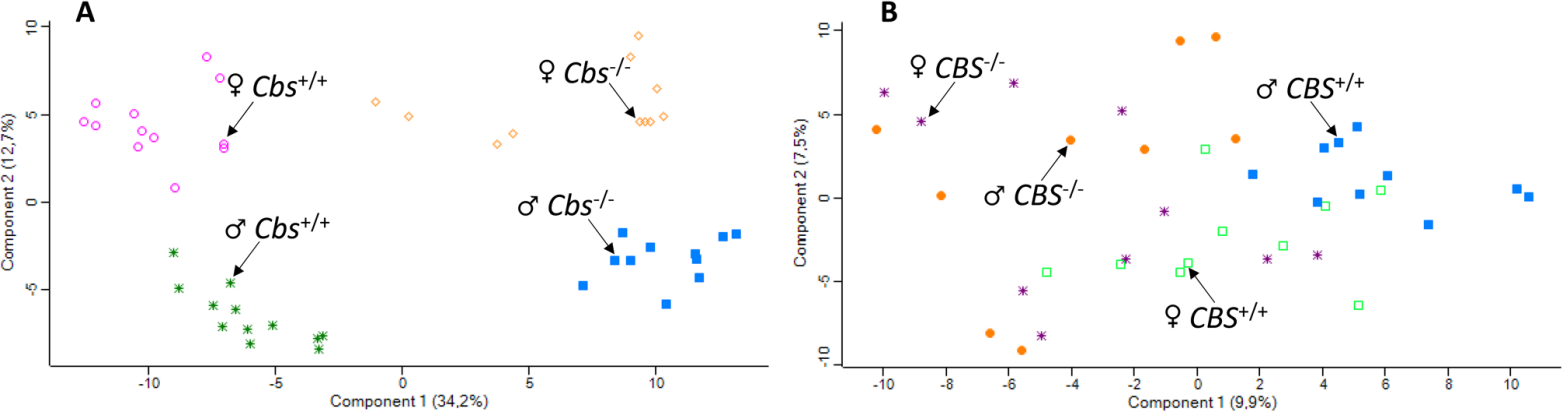
Principal component analysis of the label-free quantification intensities for plasma proteins. (**A**) Mice: *Cbs*^−/−^ female (orange diamond), *Cbs*^−/−^ male (blue square), *Cbs*^+/+^ female (purple circle), *Cbs*^+/+^ male (green star). (**B)** Humans: *CBS*^−/−^ female (purple star), *CBS* ^−/−^ male (orange filled circle), control *CBS*^+/+^ female (green square), control *CBS*^+/+^ male (blue filled square). Calculations were performed with Perseus.

To identify variations between samples in terms of global proteomic profile, principal component analysis (PCA) was carried out. There was a clear difference in PCA profiles between *Cbs*^−/−^ mice and their *Cbs*^+/+^ siblings (Fig. 1). The PCA also differentiated between males (lower clusters) and females (upper clusters) within each *Cbs* genotype. However, there was an overlap in PCA profiles between *CBS*^−/−^ patients and *CBS*^+/+^ controls (Fig. 1) and between males and females within each *CBS* genotype.

**Figure 2:**
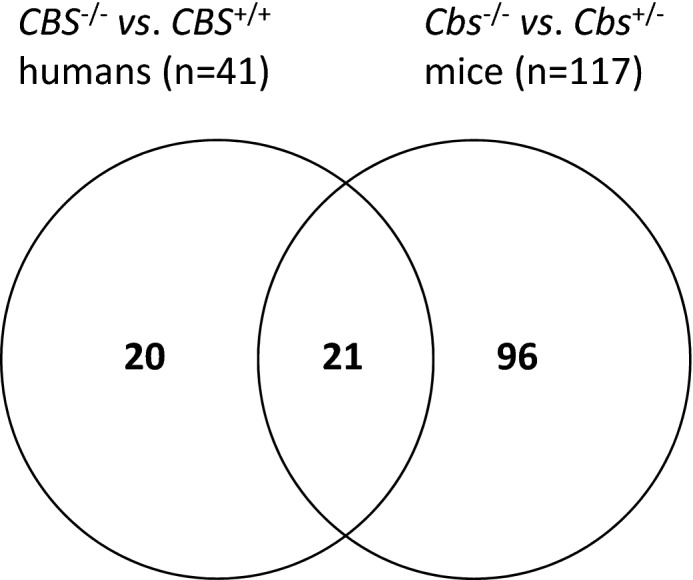
Venn diagram illustrating a partial overlap between proteins affected by CBS deficiency in mice and humans.

We identified 117 proteins differentiating between *Cbs*^−/−^ vs. *Cbs*^+/+^ in mice in the present study while 41 proteins differentiating between *CBS*^−/−^ vs. *CBS*^+/+^ were identified in humans^12^ (Fig. 2). Of these, ninety-six proteins (82%) were affected by CBS deficiency only in mice, while twenty (49%) were affected only in humans. There were twenty-one proteins (accounting for 17% and 51% of the differentiating proteins in mice and humans, respectively) that were affected by CBS deficiency in both mice and humans (Fig. 2). The differentiating proteins are listed in Supplemental Table S1.

### Plasma proteins affected by Cbs deficiency in mice

**Figure 3:**
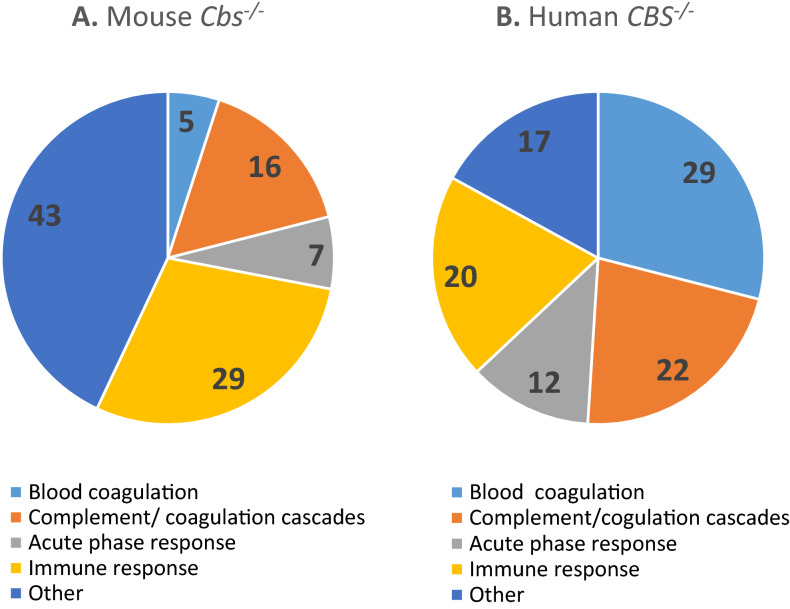
Relative numbers of proteins (%) involved in the indicated molecular processes affected in *Cbs*^−/−^ mice (**A**) and *CBS*^−/−^ patients (**B**).

Of the 117 proteins differentiating between *Cbs*^−/−^ and *Cbs*^+/+^ mice, sixty-seven (57%) were significantly upregulated (1.09- for Apoh to 7.57-fold for Ces2e) and 50 (43%) were downregulated (0.08- for Igkv4-63 to 0.89-fold for Cpn1) (Supplemental Table S1). Proteins affected by the mouse *Cbs*^−/−^ genotype are involved in blood coagulation (n = 6, 5%; ApoH, F10, Pros1, SerpinA10, SerpinC1, SerpinF2), complement/coagulation cascades (n = 19, 16%; Cpn2, Fcn1, Cpb2, Gm4788, Cfb, Klkb1, SerpinD1, SerpinG1, etc.), acute phase response (n = 8, 7%; ApoA2, Crp, Fn1, Hp, Orm1, Orm2, SerpinA3n, Ahsg), and immune response (n = 34, 29%; Apcs, Gm5571, Igkv4-63, Ighm, Igkc, etc.) (Fig. 3A, Table 1). Other proteins (n = 50, 43%) are involved in proteolytic activity (n = 9, 8%; *e.g.* Ambp, Hgfac, SerpinA1b, SerpinA1d, etc.), oxidative stress/redox homeostasis (n = 3, 2.5%; Sod3, Gpx3, Qsox1), and other processes.

**Table 1:** CBS deficiency-responsive proteins unique to, or shared between, mice and humans.

Unique to mice (n = 96)^a^			Unique to humans (n = 20)^a^	Proteins affected in mice and humans (n = 21)^b^
↑Actb	↓DnaH8	↑Mup20	↑**APOC1**	↓AFM↓
↑ApoA4	↑FetuB	↑Qsox1	↓APOC3	
↑ApoB	↑Gpld1	↑Pi16	↑**GSN**	
↑ApoD	↑H2Q10	↑Pltp		
↑ApoE	↓Hgfac	↓Pon1		
↑Azgp1	↓Igfals	↑Psap		↑**APOM**↓
↓Ces1c	↑Lcat	↑Sepp1		
Ces2e	↓Lifr	↑Serpina7		
↓Cd5l	↑Lrg1	↑Sod3		↑GC↓
↑CtsB	↑Lum	↑Thbs1		↑GPX3↑
↑Csf1r	↓Mgam	↑Vtn		
Acute phase response (n = 12): ↑Agt, ↑Apcs, ↑ApoA2, ↑Ambp, ↑Cp, ↑Crp, ↓Fn1, ↑Hp, ↑Itih3, ↑Orm1, ↑Serpina3n, ↑Tf			Acute phase response (n = 2): ↑**SAA1**, ↓**TTR**	Acute phase response (n = 3): ↑**APOA1**↓, ↑HPX↓, ↑ORM2↓
Blood coagulation (n = 4): ↑F10, ↑Serpina1b, ↑Serpina10, ↑Pros1			Blood coagulation (n = 5): ↓A1BG, ↓**F13B**, ↑FBLN1, ↓**KNG1**, ↑**SERPINA1**	Blood coagulation (n = 6): ↑**AHSG**↓, ↑APOH↓, ↑ITIH2↓, ↑SERPIND1↓, ↑**SERPINF2**↓, ↑**SERPINC1**↓
Complement/coagulation (n = 16): ↑C1qa, ↑C1qb, ↑C1qc, ↑C4b, ↓C4bp, ↑C6, ↑Cfb, ↑Cfd, ↓Cfh, ↓Cpn2, ↑Fcn-1, ↓Gm4788, ↓Mbl1; ↑Mbl2; ↑Serping1, ↓SERPINA1d			Complement/coagulation (n = 3): ↑C9, ↓**F2**, ↓FCN3	Complement/coagulation (n = 6): ↑C1R(C1ra)↓, ↑C1S(C1rs)↓, ↑CF1↓ ↑**CPB2**↑, ↓CPN1↓, ↓**KLKB1**↓,
Immune response (n = 31): ↓Igh (n = 18), ↓Igk (n = 11), Igl (n = 2)			Immune response (n = 7): ↑HEL-213, ↓**IGH@**, ↑IGHD, ↑IGHV3-7, IGHV3-72, ↑**IGK@**, ↓IGKV2D-24	Immune response (n = 2): ↑**CLU**↓, ↓IGJ↑

Proteins highlighted in bold are associated with stroke subtypes in humans (Sikora et al.^12^).

^a^Up and down arrows indicate the direction of change in protein levels.

^b^Arrows left and right to the protein acronym refer to the change in protein levels in mice and humans, respectively.

### Plasma proteins affected by CBS deficiency in humans

Of the forty-one plasma proteins differentiating between *CBS*^−/−^ and *CBS*^+/+^ humans, fifteen (37%) were significantly upregulated (from 1.11- for GSN to 3.39-fold for IGHG1) and twenty-six (63%) were significantly downregulated (from 0.57- for TTR to 0.93-fold for CLU)^12^ (Supplemental Table S1). The majority of proteins affected by the human CBS deficiency (n = 34; 83%) are involved in blood coagulation (n = 12, 29%; AHSG, APOH, F13B, FBLN1, KNG1, SERPINA1, etc.) and complement/coagulation cascades (n = 9, 22%; C9, CF1, CPB2, F2, etc.), acute phase response (n = 5, 12%; APOA1, HPX, ORM2, SAA1, TTR), and immune response (n = 8, 20%; IGHD, IGJ, APC, C4BPA, IGHV3-72, IGH@, IGK@, IGKV2D-24) (Fig. 3B, Table 1).

Of the forty-one differentially expressed proteins affected by CBS deficiency, twenty (49%) were specific to humans. The majority (n = 15, 75%) of the twenty human-specific proteins associated with CBS deficiency are involved in immune response (n = 6, 30%), blood coagulation (n = 5, 25%), complement/coagulation cascades (n = 3, 15%), and acute phase response (n = 2, 10%) (Table 1).

### Overlap between proteins affected by *CBS* genotype in mice and humans

We identified twenty-one proteins whose levels were affected *both* by the human *CBS*^−/−^ genotype and the mouse *Cbs*^−/−^ genotype (Fig. 2, Table 1). The twenty-one common proteins represented 52% and 18% of the total number of proteins affected in *CBS*^−/−^ patients and *Cbs*^−/−^ mice, respectively.

However, the majority of these common proteins (n = 15, 75%) were affected in *opposite* directions in mice and humans: fourteen proteins were upregulated in *Cbs*^−/−^ mice and downregulated in *CBS*^−/−^ humans (AHSG, APOA1, APOH, APOM, C1R, C1S, CF1, CLU, GC, HPX, ORM2, SERPINC1, SERPIND1, SERPINF2), while one was upregulated in *CBS*^−/−^ humans and downregulated in *Cbs*^−/−^ mice (IGJ). Levels of only five proteins (19%) were changed in the same direction: upregulated (CPB2, GPX3) or downregulated (AFM, CPN1, KLKB1) both in *Cbs*^−/−^ mice and *CBS*^−/−^ humans (Table 1, Supplemental Table S1).

### Organ specificity of ***Cbs***^−/−^ genotype effects on protein levels

To establish whether *Cbs*^−/−^ genotype-affected proteins identified in mouse plasma are organ specific and to identify their possible sources, we carried out label-free mass spectrometry analyses of proteomes of mouse organs. We identified fifteen proteins that were affected by Cbs deficiency both in plasma and in at least one organ; seven of those proteins were upregulated and six downregulated in the plasma and at least one organ. For the majority of proteins (13 out of 15), *Cbs*^−/−^ genotype had similar effects on protein expression in the plasma and in liver, kidney, spleen, and heart (Table 2).

**Table 2:** Relative levels of proteins affected by *Cbs*^−/−^ genotype in mouse tissues.

Protein	Tissue					
	Plasma	Liver	Kidney	Spleen	Heart	Brain
**Fold change Cbs**^−/−^**/Cbs**^**+/+**^** (P value)**						
SerpinA1d	↓0.86 (0.005)	↓0.61 (0.009)	↓0.51 (0.009)	↓0.48 (3E−04)	↓0.37 (2E−08)	
ApoE	↑2.9 (1E−20)	↑1.94 (1E−14)	↑2.4 (2E−04)	↑1.84 (4E−04)	↑1.63 (0.022)	
Ces1c	↓0.82 (0.006)	↓0.72 (0.004)			↓0.53 (1E−05)	
CtsB	↑1.5 (0.004)	↑1.6 (0.012)	↑1.6 (0.002)			
Clu	↑1.8 (3E−10)		↑2.0 (0.025)			
Gpx3	↑1.8 (2E−10)		↑1.4 (0.017)	↑1.67 (0.015)		
HP	↑7.5 (1E−10)		↑3.7 (0.017)		↑6.7 (2E−07)	
Hpx	↑1.4 (7E−06)			↓0.82 (0.032)	↓0.70 (0.018)	
Ighg2b	↓0.43 (1E−06)			↓0.20 (2E−07)		
Ighg2c	↓0.32 (9E−14)			↓0.34 (2E−07)		
Ighm	↓0.27 (7E−18)			↓0.79 (0.023)		
Igkc	↓0.45 (3E−08)		↓0.37 (7E−04)	↓0.58 (8E−04)	↓0.45 (0.001)	
Lum	↑1.4 (2E−04)		↑1.6 (0.049)			
Mup20	↑5.7 (6E−06)	↑4.6 (1F−05)	↑3.6 (9E−04)			
Tf	↑1.25 (4E−07)					↓0.68 (0.030)

Up and down arrows indicate upregulation and downregulation, respectively, of protein levels.

Specifically, SerpinA1d, a component of the complement/coagulation cascades, was downregulated in plasma, liver, kidney, spleen, and heart. Carboxylesterase Ces1c, a lipid metabolizing enzyme, was downregulated in plasma, liver, and heart. Immunoglobulins Ighg2c, Ighg2b, Ighm, and Igkc were downregulated in plasma and spleen, while Igkc was similarly downregulated in kidney and heart (Table 2).

The following proteins were upregulated: apoliprotein ApoE in plasma, liver, kidney, spleen, and heart; lysosomal antigen-processing enzyme, cathepsin CtsB in plasma, liver, and kidney; clusterin Clu in plasma and kidney; glutathione peroxidase Gpx3 in plasma, kidney, and spleen; haptoglobin Hp in plasma, kidney, and heart; Mup20 in plasma, liver, and kidney; lumican Lum in plasma and kidney (Table 2).

Levels of two proteins were changed in opposite directions in plasma and organs. An acute phase response protein, hemopexin Hpx, which was upregulated in the plasma, was downregulated in heart and spleen. An iron transport protein, serotransferrin Tf, which as upregulated in plasma, was downregulated in the brain (Table 2).

Notably, the largest number of *Cbs*^−/−^ genotype-affected plasma proteins were affected by *Cbs*^−/−^ genotype also in the kidney (n = 9), spleen (n = 8), heart (n = 6), and liver (n = 5) (Table 2). Only one protein (Tf) was affected by *Cbs*^−/−^ genotype in the brain, in addition to plasma.

### Validation of label-free mass spectrometry analysis

To validate label-free mass spectrometry experiments, we quantified five mouse and two human proteins using ELISA or Western blot assays (Table 3). We found that the acute phase response protein anti-thrombin III (SERPINC1), and the oxidative stress response protein GPX3, downregulated (0.84-fold) and upregulated (1.41-fold, *P* = 0.005), respectively, in the label-free experiments in *CBS*^−/−^ patients (Supplemental Table S1), were similarly affected by the *CBS* genotype in the ELISA assays (0.62-fold and 1.19-fold, respectively).

**Table 3:** Validation of label-free mass spectrometry data for selected proteins by ELISA and Western blotting.

*Cbs*/*CBS* genotype	Mouse					Human	
	Gpx3, ng/mL (n)	Pon1, ng/mL (n)	Serpinc1, ng/mL (n)	Mup20^a^, relative (n)	IgG^a^, relative (n)	GPX3, ng/mL (n)	SERPINC1, ng/mL (n)
^−/−^	1.7 ± 0.3 (7)	5.7 ± 1.1 (11)	66.0 ± 5.2 (6)	0.90 ± 0.66 (8)	0.31 ± 0.06 (8)	19.1 ± 4.4 (10)	455 ± 291 (10)
+ / +	1.3 ± 0.2 (9)	9.6 ± 3.6 (10)	55.3 ± 5.1 (5)	0.54 ± 0.73 (8)	1.00 ± 0.14 (8)	16.1 ± 1.5 (10)	723 ± 209 (14)
Fold change	1.32	0.60	1.19	1.67	0.31	1.19	0.63
*P* value^b^	0.0035	0.005	0.019	0.004^b^	4E−06	< 0.05	< 0.05

Quantified by ELISA.

^a^Quantified by Western blotting. ^b^Calculated using Log transformed data. ^b^Two-sided *t*-test.

Mouse Serpinc1, upregulated in *Cbs*^−/−^ mice (1.34-fold, *P* = 7E−06; Supplemental Table S1) in the label-free experiments was also found to be upregulated in the ELISA assays (1.19-fold, *P* = 0.019; Table 3). Mup20, which was upregulated in *Cbs*^−/−^ mice in the label-free experiments (5.75-fold, *P* = 6E−06; Supplemental Table S1) was also upregulated in the Western blot assays (1.67-fold, *P* = 0.004; Table 3). IgGs that were downregulated in *Cbs*^−/−^ mice in the label-free experiments (0.43- to 0.32-fold, *P* < 1E−06; Supplemental Table S1) were similarly downregulated in the Western blot assays (0.31-fold, *P* = 4E−06; Table 3).

### Bioinformatics analysis

To identify biological pathways linked to proteins affected by Cbs deficiency in mice and CBS deficiency in humans, we performed bioinformatics analyses using IPA resources. Analyses for the human CBS deficiency have been described before^12^. We have found that in the mouse Cbs deficiency, proteins from 19 pathways were overrepresented among differentially expressed proteins, while proteins from 17 pathways were enriched in the human.

**Figure 4:**
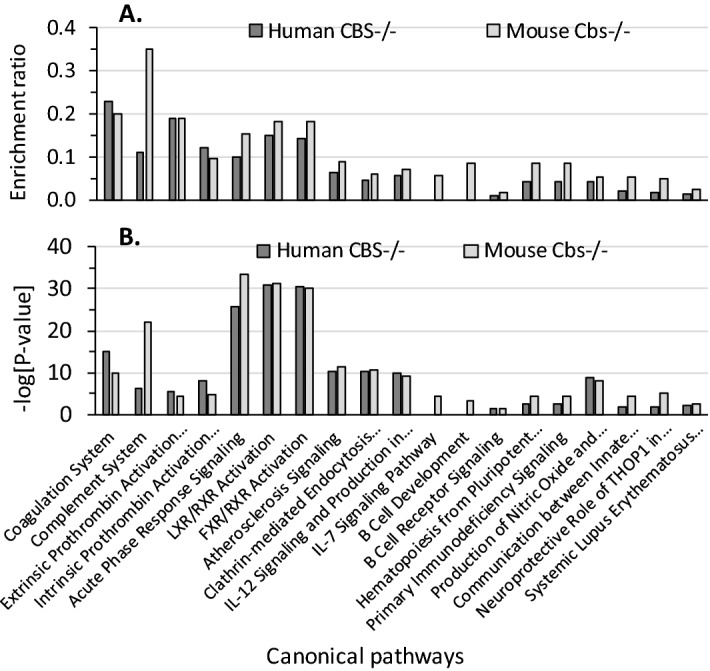
Enrichment ratios (**A**) and canonical pathways (**B**) for differentially expressed proteins in *CBS*^−/−^ human patients and *Cbs*^−/−^ mice identified by IPA. Benjamini-Hochberg, Benferroni, and false discovery rate corrections were applied to minimize the number of false positives.

CBS deficiency (Fig. 4A). The majority of those pathways affect directly or indirectly blood clotting.

Overall, enrichment ratios (defined as the number of observed proteins divided by the number of expected proteins from each pathway category) were higher in *Cbs*^−/−^ mice than in *CBS*^−/−^ patients, except for the Coagulation System pathway, which had a higher enrichment ratio in *CBS*^−/−^ patients (0.23, − log[*p*-value] = 15.1) than in *Cbs*^−/−^ mice (0.20, − log[*p*-value] = 10.0). The Complement System pathway had the highest enrichment ratio in *Cbs*^−/−^ mice (0.35, − log[*p*-value] = 21.9), three-fold higher than in *CBS*^−/−^ patients (0.11, − log[*p*-value] = 6.3). Two pathways, IL-7 Signaling and B Cell Signaling, contained proteins significantly enriched in only in *Cbs*^−/−^ mice (Fig. 4A).

The top five highly significant pathways affected by both mouse Cbs deficiency and human CBS deficiency were Acute Phase Response Signaling (− log[*p*-value] = 25.9 [humans] and 33.5 [mice]), LXR/RXR Activation (− log[*p*-value] = 30.8 [humans] and 31.4 [mice]), FXR/RXR Activation (− log[*p*-value] = 30.5 [humans] and 30.0 [mice]), Coagulation System, and Complement System (Fig. 4B).

### Cbs-deficient mice

**Figure 5:**
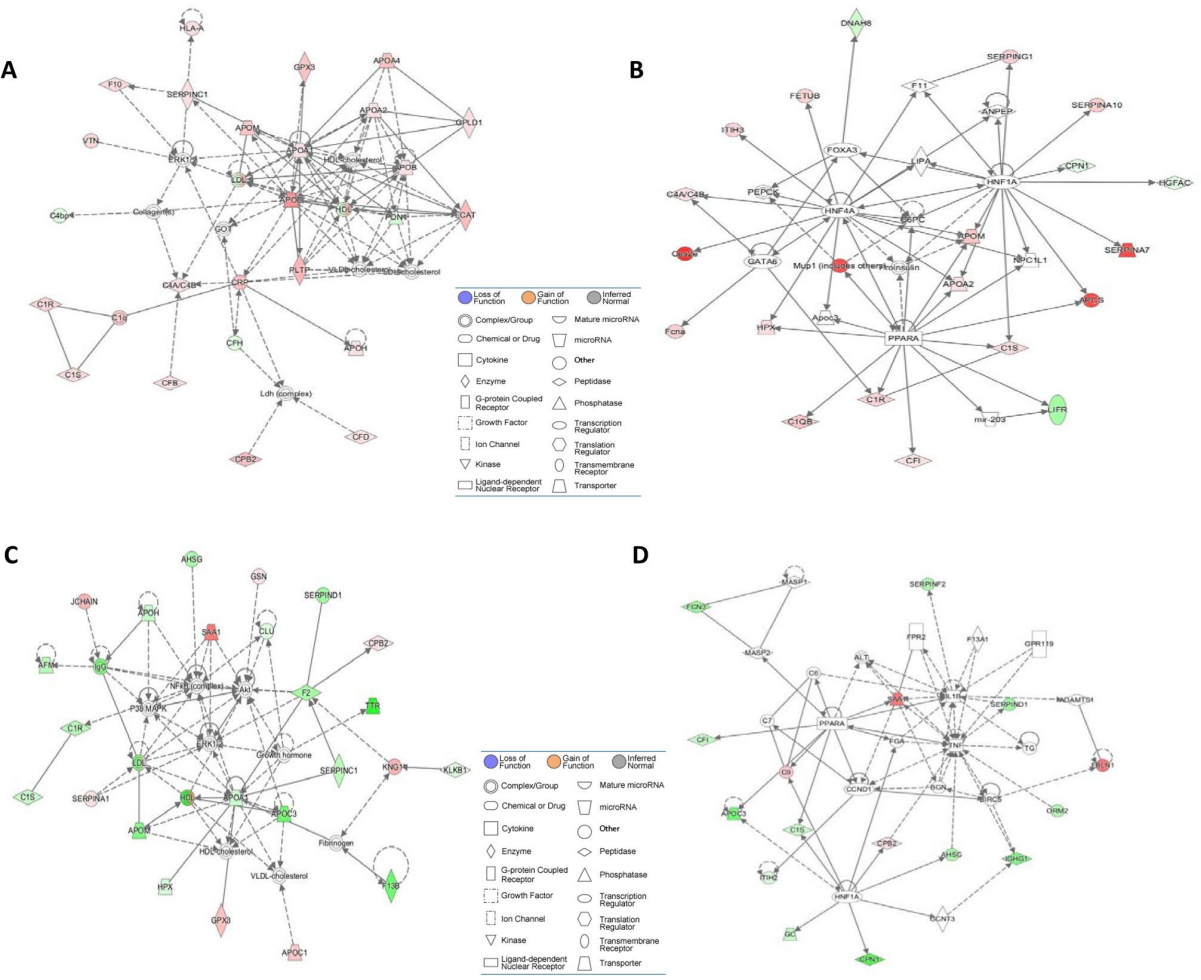
Top molecular networks of CBS deficiency-responsive proteins. Mice: (**A**) Protein Synthesis, Cardiovascular Disease, Hematological Disease; (**B**) Developmental Disorder, Hereditary Disorder, Immunological Disease. Humans: (**C**) Metabolic Disease, Hematological System Development and Function, Lipid Metabolism; (**D**) Humoral Immune Response, Inflammatory Response, Developmental Disorder. Upregulated proteins are highlighted in red, and downregulated proteins are highlighted in green. Molecules in these networks, scores, and associated diseases and functions are listed in Table 4. Drawing shown in panel (**C**) is from Sikora et al.^12^.

IPA identified five biological networks associated with mouse Cbs deficiency (Table 4). The two top-scoring networks were Protein Synthesis, Cardiovascular Disease, Hematological Disease and Developmental Disorder, Hereditary Disorder, Immunological Disease. The Protein Synthesis, Cardiovascular Disease, Hematological Disease network, with a score of 50, contains 35 proteins: 25 were quantified by the label-free mass spectrometry and 10 were identified by IPA to interact with the quantified proteins (Fig. 5A). This network involves proteins participating in lipid metabolism and the complement/coagulation cascades that interact with Erk1 and Crp. The Developmental Disorder, Hereditary Disorder, Immunological Disease network, with a score of 40, contains 35 proteins: 21 quantified by the label-free mass spectrometry and 14 identified by IPA to interact with the quantified proteins (Fig. 5B). This network contains proteins of the complement/coagulation cascades that strongly interact with regulators of lipid metabolism (Ppara) and gene expression in the liver (Hnf1a, Hnf4a).

**Table 4.**
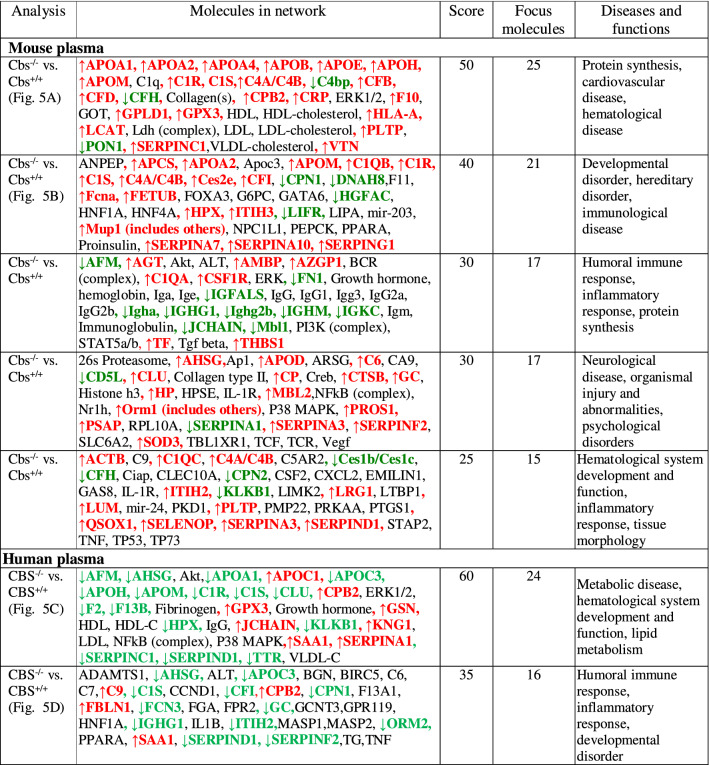
Top molecular networks of CBS deficiency-responsive human and mouse proteins. Upregulated ( ) and downregulated ( ) proteins are highlighted in red and green, respectively. Graphical illustrations of interactions between proteins in these networks are shown in Fig. 5A–D. Data for human plasma are from Sikora et al.^12^.

### CBS-deficient patients

IPA identified two biological networks associated with human CBS deficiency^12^ (Table 4). The Humoral Immune Response, Inflammatory Response network, with a score of 60, contains 34 proteins: 24 quantified by label-free mass spectrometry and 10 identified by IPA to interact with the quantified proteins (Fig. 5C). Proteins in this network show strong interactions centering on MAP/ERK1, NF-κ-B, and APOA1/HDL. The Metabolic Disease, Hematological System Development and Function, Lipid Metabolism, with a score of 35, contains 35 proteins: 16 quantified by label-free mass spectrometry and 19 identified by IPA to interact with the quantified proteins (Fig. 5D). Proteins in this network show strong interactions centering on the proinflammatory cytokines IL-1B and TNF.

## Discussion

In this comparative proteomic study, we found that thrombosis-susceptible *CBS*^−/−^ patients and thrombosis-resistant *Cbs*^−/−^ mice exhibit different expression patterns of proteins involved in the blood coagulation, complement/coagulation cascades, and acute activation and immune responses. Notably, proteins involved in the blood coagulation and complement/coagulation cascades represented a greater fraction of proteins affected in *CBS*^−/−^ patients (51%) than in *Cbs*^−/−^ mice (21%). Further, although some proteins were affected in both *CBS*^−/−^ patients and *Cbs*^−/−^ mice, these common proteins we affected in *opposite* directions in the two species. Our findings suggest that species-specific differences in the effects of CBS deficiency on these processes could account for the pro-thrombotic phenotype of *CBS*^−/−^ patients and the thrombosis-resistant phenotype of *Cbs*^−/−^ mice. Comparative proteomics of CBS deficiency have not been studied before, and, to our best knowledge, this is the first proteomic study of disparately different thrombotic phenotypes in humans and mice. Overall, our findings suggest that *Cbs*^−/−^ mice have a better adaptive response to protect from prothrombotic effects of hyperhomocysteinemia than humans. Discussion of specific changes in the proteomes of human *CBS*^−/−^ patients and *Cbs*^−/−^ mice identified in the present work and their roles in blood coagulation follows below.

Our findings show that KNG1 protein levels are reduced in *CBS*^−/−^ patients, but unaffected in *Cbs*^−/−^ mice. KNG1 is an inhibitor of thiol proteases that inhibits the thrombin- and plasmin-induced aggregation of thrombocytes (https://www.nextprot.org/entry/NX_P01042/). Variants in the *KNG1* gene are associated with venous thrombosis^15^. Thus, reduced KNG1 levels could contribute to the pro-thrombotic phenotype observed in *CBS*^−/−^ patients^1,4,9,10^.

The anti-thrombin SERPINC1, genetic variants of which are associated with venous thrombosis^15^, was downregulated in *CBS*^−/−^ humans and upregulated in *Cbs*^−/−^ mice. The downregulation of the anti-thrombin SERPINC1 would increase thrombin activity (and thus increase blood clotting) in *CBS*^−/−^ patients. In contrast, the upregulation of the anti-thrombin Serpinc1 would reduce thrombin activity (and thus reduce blood clotting) in *Cbs*^−/−^ mice. These findings suggest that the changes in levels of components regulating thrombin activity contribute to the thrombotic phenotype in *CBS*^−/−^ patients and the absence of thrombosis in *Cbs*^−/−^ mice^7,8^.

We found that SERPIND1, heparin cofactor 2, was downregulated in *CBS*^−/−^ patients and upregulated in *Cbs*^−/−^ mice (Table 1, Supplemental Table S1). Because SERPIND1 deficiency can lead to increased thrombin generation and a hypercoagulable state^16^, these findings could account for the thrombotic phenotype of *CBS*^−/−^ patients and thrombosis-resistance of *Cbs*^−/−^ mice.

APOH is a component of an important anticoagulation pathway. It inhibits the contact-activated intrinsic blood coagulation pathway by binding phospholipid at the surface of damaged cells^17^, von Willebrand factor‐dependent platelet adhesion and aggregation^18^, and the pro-coagulation activities of thrombin^19^. Polymorphisms in the *APOH* gene that affect these functions, are risk factors for venous thrombosis in a Chinese population^20^. The present findings that APOH is downregulated in *CBS*^−/−^ patients and upregulated in *Cbs*^−/−^ mice (Table 1, Supplementary Table S1), could contribute to the thrombotic phenotype of *CBS*^−/−^ patients and the absence of thrombosis in *Cbs*^−/−^ mice.

The present study found that the acute phase protein Orm2 was one of the most highly (4.95-fold) upregulated proteins in *Cbs*^−/−^ mice, while ORM2 was significantly downregulated (0.8-fold) in *CBS*^−/−^ patients (Table 1, Supplemental Table S1). Because of its effects on inflammation, ORM2 has been suggested to have a protective effect on stroke in humans. In mice Orm2 has been show to protect from ischemic stroke: levels of Orm2 increase in a mouse model of ischemic stroke and administration of the Orm2 protein significantly decreases infarct size and neurological deficit score^21^. Thus, our present findings, in conjunction with the mouse Orm2 study, suggest that the high upregulation of Orm2 would protect *Cbs*^−/−^ mice from ischemic stroke, and that this protective response is not induced in *CBS*^−/−^ patients.

Although previous findings show that *Cbs*^−/−^ mice do not have a thrombotic phenotype^7,8^ found in *CBS*^−/−^ patients^1,4,9,10^, severe stroke phenotype can be induced in homozygous *Cbs*^−/−^ mice by the middle cerebral artery ischemia/reperfusion injury^22^. A similar stroke phenotype can also be induced in heterozygous *Cbs*^+/−^ mice, which have only slightly elevated plasma Hcy levels, 6.1 vs. 3.1 μM. Surprisingly, treatment with the *N*-methyl-D-aspartate receptor antagonist memantine protects *Cbs*^+/−^ but not *Cbs*^−/−^ mice from experimental stroke mice. This has been suggested to be due to an increased expression of Hcy-insensitive *N*-methyl-D-aspartate receptor subunit NR2B in *Cbs*^−/−^ mice. However, it is unclear whether activation of *N*-methyl-D-aspartate receptors contributes to the experimental stroke phenotype of *Cbs*^−/−^ mice^22^.

We also found that several proteins of the Coagulation System, such as Serpina1b, Serpina10, and the anticoagulant protein Pros1, which are known to inhibit thrombin activity or its generation, were upregulated in *Cbs*^−/−^ mice, but not in *CBS*^−/−^ patients (Table 1, Supplementary Table S1). Pros1 inhibits thrombin generation by binding and inhibiting FXa and FVa in the prothrombinase complex^23^, Serpina1b inhibits plasmin and thrombin, while Serpina10, a negative regulator of blood coagulation, inhibits the activated coagulation factors X and XI^24^. These findings suggest reduced propensity to clotting in *Cbs*^−/−^ mice, consistent with absence of thrombosis in these animals^7,8^.

In addition to the Coagulation System, we found that proteins affected by CBS deficiency both in humans and mice were significantly enriched in other molecular pathways, many of which directly or indirectly affect blood clotting (Fig. 4). For example, one of the CBS deficiency-responsive pathways—LXR/RXR Activation—involves RXR, which has a negative regulatory role in platelet function/aggregation and thrombus formation^25^. Notably, we identified the LXR/RXR pathway proteins that were downregulated in *CBS*^−/−^ patients but upregulated in *Cbs*^−/−^ mice (AHSG, APOA1, APOH, APOM, CLU, GC, HPX, SERPINF2) (Table 1, Supplementary Table S1), indicating opposite regulation of the expression/function of those proteins in *Cbs*^−/−^ mice and *CBS*^−/−^ humans. One of those proteins, AHSG, a potent anti-inflammatory cytokine^26^, is known to be involved in platelet degranulation (https://plateletweb.bioapps.biozentrum.uni-wuerzburg.de/), while SERPINF2 is a serine protease inhibitor responsible for inactivating plasmin^27^. As treatments with RXR ligands have cardioprotective effects^25^, our findings may offer a similar strategy for the prevention of thrombosis in *CBS*^−/−^ patients.

We found that the Complement System had the highest enrichment ratio in *Cbs*^−/−^ mice, three-fold higher than in *CBS*^−/−^ patients (Fig. 4). We identified sixteen proteins of the complement in *Cbs*^−/−^ mice and four in *CBS*^−/−^ patients (Table 1, Supplementary Table S1). Of the four complement proteins affected in *CBS*^−/−^ patients (C1R, C1S, C9, CFI), three were also affected in *Cbs*^−/−^ mice (C1R, C1S, CFI). Notably, while C1R, C1S, CFI were upregulated in *Cbs*^−/−^ mice, they were downregulated in *CBS*^−/−^ patients.

We found that Il-7 signaling pathway was affected in *Cbs*^−/−^ mice. IL-7 plays an important role in the development and proliferation of B and T cells in mouse and T cells in humans by activating the JAK-STAT PI-3 kinase and the Src kinase pathways. *Cbs*^−/−^ mice showed significantly downregulated expression of immunoglobulins such as Igha, Ighm, Ighg (1, 2c, 2b), and Ighv (1-62-2, 1-76, 1-81, 1-82, 5-17, 6-3, 9-3, 10-1) (Table 1, Supplementary Table S1), which suggests attenuated immune response due to reduced Il-7/B-cell signaling. In contrast, *CBS*^−/−^ patients had significantly upregulated expression of IGHD, IGHV 3-7, and IGHV 3-72, which suggests enhanced immune response.

The interconnections between the blood coagulation and complement cascades have been observed in various clinical and experimental settings^28^. Here, using the label-free mass spectrometry we have identified such interconnections in *CBS*^−/−^ patients and *Cbs*^−/−^ mice and shown that CBS deficiency is characterized by complement-associated inflammatory responses. We have also identified specific changes in the levels of protein components of the blood coagulation system that could provide an explanation for the thrombotic phenotype in *CBS*^−/−^ patients and the absence of thrombosis in *Cbs*^−/−^ mice. These findings also suggest that ORM2, F10, SOD3, HP, SERPING1, SERPINA10, SERPINA3N, SERPINA7, and/or CES2E proteins might be targeted for potential anti-thrombotic interventions in humans.

## Materials and methods

### CBS-deficient patients

The study included plasma samples from the Danish^29^ (n = 4) and Polish^30,31^ (n = 6) CBS-deficient patients (mean age 37.3 ± 7.3 years, 5 males, 5 females) and gender- and age-matched healthy control individuals (n = 14). Characteristics of the CBS-deficient patients and controls have been described^12^. Briefly, two of the Polish CBS-deficient patients had a stroke before diagnosis in childhood at age 3 to 15. Danish CBS-deficient patients were diagnosed at age 7, 21, 22, and 36; two of them had episodic transient ischemic attacks at age 22 and 36. Patients were on a Hcy-lowering vitamin B_6_ treatment, except for the six vitamin B_6_ non-responsive patients who were treated with betaine.

All experimental protocols conformed to the Ethical Guidelines of the World Medical Association Declaration of Helsinki and was approved by the Bioethics Committee of the University of Medical Sciences, Poznań, Poland (decision no. 661/16). Written informed consent was obtained from all participants or legal guardians. The procedures followed were in accordance with institutional guidelines and regulations.

### Human plasma samples

Blood was drawn by venous puncture during routine health examination visits at doctor’s office as previously described^12^. Blood samples were collected into EDTA tubes, plasma was separated by centrifugation (2,000 g, 15 min, 4 °C), collected, and stored at − 80 °C.

### Cbs-deficient mice

Transgenic *Tg-I278T Cbs*^+/−^ mice on C57BL/6 J genetic background^7^ were mated to generate *Tg-I278T Cbs*^−/−^ and *Tg-I278T Cbs*^+/+^ animals. The mice were bred and housed at the New Jersey Medical School Animal Facility^32,33^. In these animals, the human *CBS-I278T* variant is under control of the zinc-inducible metallothionein promoter, which prevents the neonatal lethality of the mouse *Cbs*^−/−^ genotype by supplementation of the drinking water with 25 mM ZnCl_2_^7^. The zinc water is replaced by plain water after weaning at 30 days. The mice were fed a normal rodent chow (LabDiet5010, Purina Mills International, St. Louis, MO). *Cbs* genotype was established by PCR. *Cbs*^−/−^ and *Cbs*^+/+^ mice of both sexes (8-month-old, 12/group, sex-matched), were used in experiments. Animal procedures were approved by the Institutional Animal Care and Use Committee at the New Jersey Medical School. All experiments were carried out in accordance with relevant guidelines and regulations.

### Blood and tissue collection and processing

Blood was collected from the cheek vein into Eppendorf tubes containing 1% (v/v) 0.5 M EDTA. After centrifugation (2,000 g, 15 min, 4 °C), separated plasma and cells were frozen at − 80 °C. Mice were euthanized using CO_2_, organs collected and frozen on dry ice. Liver, kidneys, spleen, and brain were powdered with dry ice using mortar and pestle, and stored at − 80 °C. Proteins were extracted from powdered tissues using triethylammonium bicarbonate pH 8.5 buffer, 0.1% SDS (Millipore-Sigma) with sonication (Bandelin SONOPLUS HD 2070) on wet ice (five 30-s strokes with 1 min cooling interval after each stroke). Extracts were clarified by centrifugation (15,000 g, 5 min, 4 °C), protein was precipitated with acetone (40 min, − 20 °C), the protein pellet collected by centrifugation (15,000 g, 10 min, 4 °C), and dissolved by sonication in 50 mM ammonium bicarbonate. Protein concentration was quantified using a 2-D Quant kit (GE Healthcare).

### Digestion with trypsin

Ten-microgram aliquots of protein were diluted with 50 mM ammonium bicarbonate, reduced with 5.5 mM dithiothreitol (5 min, 95ºC), and alkylated with 5 mM iodoacetamide (20 min in the dark, 25 °C). Protein samples were digested with Promega sequencing-grade trypsin (0.2 µg, overnight, 37 °C)^12^.

### Label-free mass spectrometry

Dionex UltiMate 3,000 RSLC nanoLC System attached to Q Exactive Orbitrap mass spectrometer (Thermo Fisher Scientific) was used for analyses as previously described^12^. Tryptic peptides were separated on a C18 column Acclaim PepMap RSLC nanoViper (75 µm × 25 cm, 2 µm) using 4–60% acetonitrile gradient, 0.1% formic acid (300nL/min, 30 °C, 230 min). Mass spectra were acquired on the Q Exactive in a data-dependent mode using top 10 data-dependent MS/MS scans. Target value for the full scan MS spectra was 1 × 10^6^ with a maximum injection time of 100 ms and a resolution of 70,000 at m/z 400. The MS scan range was 300–2000 m/z. Ten most intense ions charged two or more were selected with a 2-Da isolation window and fragmented by higher energy collisional dissociation with NCE 25. The ion target value for MS/MS was 5 × 10^4^ with a maximum injection time of 100 ms and a resolution of 17,500 at m/z 400.

### Data analysis

Proteins were identified using UniProt human database with a precision tolerance 10 ppm for peptide masses and 0.08 Da for fragment ion masses as previously described^12^. Two missed trypsin cleavages were allowed. S-carbamidomethyl-Cys was a fixed modification and Met-sulfoxide was allowed as a variable modification. For protein identification/quantification, each dataset was imported into MaxQuant 1.5.3.30 version (MPIB, Martinsried, Germany). Protein was positively identified if at least two peptides were found by the Andromeda search engine, and a peptide score had a false rate of discovery of 0.01. The analyses were based on label-free quantification (LFQ) of peptide intensities. The MaxQuant data were filtered for reverse identifications (false positives), contaminants, and proteins “only identified by site”. The mean LFQ intensities + /− standard deviation were calculated for each identified differentially expressed protein. The fold change values (FC) in the differentiating protein levels were assessed by comparing the mean LFQ intensities among experimental groups.

### Statistics

The data were analyzed using Perseus ver. 1.5.3.2 software (part of MaxQuant package) as previously described^12^. Numeric data were log-transformed and samples were annotated with their group affiliation. Data were filtered—proteins with valid values in 70% of samples in at least one group were kept in the table. For multiple comparisons, one-way analysis of variance (ANOVA) with a Bonferroni correction for multiple testing was performed. Two-sample t-test was used for comparisons between two groups with *P* values < 0.05 used for truncation. The resulting lists of differentiating proteins were normalized using Z-score algorithm for hierarchical clustering of data. Multivariate analyses were carried out by untargeted principal component analysis (PCA)^12^.

### Bioinformatics analysis

Proteins that were quantified as unique and non-redundant were used in analyses. Proteins were considered to be differentially expressed if the FC difference was statistically significant (*P* < 0.05). The proteins were considered as differentiating if it was quantified by at least two peptides with > 99% confidence. Uncharacterized proteins were excluded from the analysis.

Bioinformatic analysis to discover biological pathways and networks for proteins affected by the *Cbs*^−/−^ genotype were performed out using the Ingenuity Pathway Analysis software (IPA, Ingenuity Systems, Mountain View, CA). The data sets containing differentially expressed proteins were uploaded into the IPA Knowledge database to map proteins and metabolites to global molecular networks and identify their interactions with other proteins in the database.

### ELISA and western blot assays

To validate differentially expressed proteins, we quantified human SERPINC1 and GPX3, mouse Gpx3, Pon1, and Serpinc1 using commercial ELISA kits (FineBiotech and AssayPro). Triplicate assays were performed following the manufacturers’ protocols. A_450_ was read using microplate reader (Infinite M200 Pro, Tecan, Switzerland). Mouse Mup20 and IgG were quantified by Western blotting using commercial anti-Mup and anti-IgG antibodies.

### Homocysteine assays

Mouse and human plasma total Hcy was quantified by an HPLC-based method as previously described^14^.

## Supplementary information

Supplementary information.
