# Supplementary material for: Cystathionine β-synthase deficiency: different changes in proteomes of thrombosis-resistant Cbs−/− mice and thrombosis-prone CBS−/− humans
Source: Sci Rep. 2020 Jul 1;10:10726. doi: 10.1038/s41598-020-67672-5 (PMC7329814; doi:10.1038/s41598-020-67672-5)
Supplement: Supplementary file 1 — Supplementary information. [file 41598_2020_67672_MOESM1_ESM.docx]

**Cystathionine β-Synthase Deficiency: Different Changes in Proteomes of Thrombosis-resistant *Cbs*^-/-^ Mice and Thrombosis-prone *CBS*^-/-^ Humans**

**Marta Sikora, Izabela Lewandowska, Łukasz Marczak, Ewa Bretes, Hieronim Jakubowski**

**Supplementary Information**

| **Supplementary Table S1.** CBS deficiency-responsive proteins in humans and mice. | | | | | | | |
| --- | --- | --- | --- | --- | --- | --- | --- |
| **Protein name** | **Human plasma*** | | | **Mouse plasma** | | | **Pathway/Process** |
|  | **Gene name** | **Fold change**  ***CBS*^-/-^/*CBS*^+/+^** | ***P*-value** | **Gene name** | **Fold change**  ***Cbs*^-/-^/*Cbs*^+/+^** | ***P*-value** |  |
| Actin, cytoplasmic 1 |  |  |  | Actb | 1.31 | 0.004 | ATP-dependent chromatin remodeling; Platelet aggregation |
| Afamin | *AFM* | 0.83 | 0.007 | *Afm* | 0.73 | 3E-06 | Vitamin transport |
| Alpha-1-acid glycoprotein 1 |  |  |  | *Orm1* | 1.62 | 3E-07 | Acute-phase response |
| Alpha-1-acid glycoprotein 2 | *ORM2* | 0.81 | 0.042 | *Orm2* | 4.95 | 5E-05 | Acute-phase response |
| Alpha-1-antitrypsin | *SERPINA1* | 1.14 | 0.045 |  |  |  | Acute-phase response; Blood coagulation |
| Alpha-1-antitrypsin 1-2 |  |  |  | *Serpina1b* | 1.11 | 0.033 | Serine proteases inhibitor (elastase, plasmin, thrombin) |
| Alpha-1-antitrypsin 1-4 |  |  |  | *Serpina1d* | 0.86 | 0.005 | Negative regulation of endopeptidase activity (trypsin, chymotrypsin), complement/cogulation cascades |
| Alpha-1B-glycoprotein | *A1BG* | 0.90 | 0.034 |  |  |  | Neutrophil/platelet degranulation |
| Alpha-2-antiplasmin | *SERPINF2* | 0.90 | 1E-05 | *Serpinf2* | 1.22 | 0.032 | Negative regulator of fibrinolysis |
| Alpha-2-HS-glycoprotein | *AHSG* | 0.80 | 1E-04 | *Ahsg* | 1.17 | 2E-04 | Acute-phase response |
| Angiotensinogen |  |  |  | *Agt* | 1.27 | 2E-04 | Hypertrophic cardiomyopathy; Renin-angiotensin system; Vascular smooth muscle contraction; Blood pressure regulation |
| Antithrombin-III | *SERPINC1* | 0.84 | 4E-05 | *Serpinc1* | 1.34 | 7E-06 | Blood coagulation; inhibits thrombin, matriptase-3/TMPRSS7, factors IXa, Xa, XIa. |
| Apolipoprotein A-I | *APOA1* | 0.87 | 0.038 | *Apoa1* | 1.35 | 8E-05 | Fat digestion and absorption |
| Apolipoprotein A-II |  |  |  | *Apoa2* | 1.24 | 0.034 | Acute inflammatory response |
| Apolipoprotein A-IV |  |  |  | *Apoa4* | 1.87 | 2E-12 | Fat digestion and absorption; Vitamin digestion and absorption |
| Apolipoprotein B-100 |  |  |  | *Apob* | 1.21 | 0.017 | Cholesterol homeostasis; Fat digestion and absorption |
| Apolipoprotein C-I | *APOC1* | 1.40 | 0.032 |  |  |  | Lipid transport/metabolism |
| Apolipoprotein C-III | *APOC3* | 0.68 | 0.014 |  |  |  | Lipid transport/metabolism |
| Apolipoprotein D |  |  |  | *Apod* | 1.26 | 0.023 | Aging; Brain development |
| Apolipoprotein E |  |  |  | *Apoe* | 2.91 | 1E-20 | Alzheimer's disease |
| Apolipoprotein M | *APOM* | 0.70 | 0.001 | *Apom* | 1.75 | 0.028 | Cholesterol transport |
| Beta-2-glycoprotein 1 | *APOH* | 0.86 | 0.015 | *Apoh* | **1.09** | 0.043 | May prevent intrinsic blood coagulation by binding phospholipid at the surface of damaged cells; |
| C4b-binding protein |  |  |  | *C4bp* | 0.75 | 0.006 | Complement/coagulation cascades |
| Carboxylesterase 1C |  |  |  | *Ces1c* | 0.82 | 0.006 | Lipid catabolic procress (sterol esters, triglycerides) |
| Carboxypeptidase B2 | *CPB2* | 1.12 | 0.044 | *Cpb2* | 1.96 | 1E-11 | Complement/coagulation cascades; Pancreatic secretion; Protein digestion and absorption |
| Carboxypeptidase N catalytic chain | *CPN1* | 0.66 | 0.004 | *Cpn1* | 0.89 | 0.010 | Bradykinin catabolic process; Insulin processing |
| Carboxypeptidase N subunit 2 |  |  |  | *Cpn2* | 0.75 | 1E-10 | Regulation of Complement cascade |
| Cathepsin B |  |  |  | *Ctsb* | 1.51 | 0.004 | Antigen processing and presentation; Lysosome |
| CD5 antigen-like |  |  |  | *Cd5l* | 0.39 | 1E-15 | Apoptosis, inflammatory response |
| Ceruloplasmin |  |  |  | *Cp* | 1.11 | 0.012 | Porphyrin and chlorophyll metabolism |
| Clusterin | *CLU* | 0.93 | 0.019 | *Clu* | 1.76 | 3E-10 | Chaperone-mediated protein folding; immune complex clearance; inhibits amyloid formation |
| Coagulation factor X |  |  |  | *F10* | 1.46 | 2E-07 | Blood coagulation; converts pro- to thrombin in the presence of F5A |
| Coagulation factor XIII B-chain | *F13B* | 0.69 | 0.000 |  |  |  | Blood coagulation; fibrin-stabilizing factor; negative regul. of cell protein catabolic process |
| Complement C1q subunit A |  |  |  | *C1qa* | 1.83 | 6E-04 | Complement/coagulation cascades |
| Complement C1q subunit B |  |  |  | *C1qb* | 1.79 | 0.003 | Complement/coagulation cascades |
| Complement C1q subunit C |  |  |  | *C1qc* | 1.64 | 0.020 | Complement/coagulation cascades |
| Complement C1R subcomponent | *C1R* | 0.82 | 5E-05 | *C1ra* | 1.53 | 0.002 | Complement/coagulation cascades |
| Complement C1S subcomponent | *C1S* | 0.86 | 0.004 | *C1sa* | 1.45 | 9E-05 | Complement/coagulation cascades |
| Complement C4-B |  |  |  | *C4b* | 1.33 | 8E-06 | Complement/coagulation cascades |
| Complement factor B |  |  |  | *Cfb* | 1.43 | 8E-13 | Complement/coagulation cascades |
| Complement factor D |  |  |  | *Cfd* | 1.33 | 0.021 | Complement/coagulation cascades |
| Complement factor H |  |  |  | *Cfh* | 0.79 | 0.007 | Complement/coagulation cascades |
| Complement factor 9 | *C9* | 1.36 | 0.004 |  |  |  | Complement/coagulation cascades |
| Complement factor I | *CF1* | 0.85 | 0.002 | *Cfi* | 1.14 | 1E-04 | Complement/coagulation cascades |
| C-reactive protein |  |  |  | *Crp* | 1.90 | 6E-04 | Acute inflammatory; acute-phase response |
| Dynein heavy chain 8, axonemal |  |  |  | *Dnah8* | 0.57 | 0.026 | Cellular component movement |
| Epididymis luminal protein 213 | *HEL-213* | 1.55 | 0.003 |  |  |  | Immune response |
| Extracellular Cu-Zn superoxide dismutase |  |  |  | *Sod3* | 4.46 | 8E-10 | Response to oxidative stress |
| Fetuin-B |  |  |  | *Fetub* | 1.54 | 6E-10 | Egg fertilization; Sperm binding; Inhibits Cys-endopeptidase activity |
| Fibronectin |  |  |  | *Fn1* | 0.87 | 7E-05 | Acute-phase response; Angiogenesis |
| Fibulin-1 | *FBLN1* | 2.13 | 0.001 |  |  |  | Blood coagulation/fibrin clot form. |
| Ficolin-3 | *FCN3* | 0.76 | 0.003 |  |  |  | Complement(lectin)/immune response activation |
| Ficolin-1 |  |  |  | *Fcn1* | 1.67 | 8E-05 | Complement(lectin)/immune response activation |
| Gelsolin | *GSN* | 1.11 | 0.015 |  |  |  | Actin filament capping; Amyloid fibril formation |
| Glutathione peroxidase 3 | *GPX3* | 1.41 | 0.005 | *Gpx3* | 1.77 | 2E-10 | Response to oxidative stress |
| H-2 class I histocompatibility antigen, Q10 alpha chain |  |  |  | *H2-Q10* | 1.31 | 0.002 | Adaptive immune response |
| Haptoglobin |  |  |  | *Hp* | 7.51 | 1E-08 | Acute phase response |
| Hemopexin | *HPX* | 0.90 | 0.004 | *Hpx* | 1.42 | 7E-06 | Cellular iron ion homeostasis |
| Heparin cofactor 2 | *SERPIND1* | 0.77 | 0.005 | *Serpind1* | 1.46 | 3E-09 | Complement/coagulation cascades |
| Hepatocyte growth factor activator |  |  |  | *Hgfac* | 0.77 | 0.003 | Serine-type endopeptidase activity |
| Ig alpha chain C region |  |  |  | *Igha* | 0.55 | 2E-04 | Immune response |
| IGH@ protein | *IGH@* | 0.71 | 0.006 |  |  |  | Immune response |
| Ig heavy chain V-III region GAL | *IGHV3-7* | 1.64 | 0.019 |  |  |  | Immune response |
| Ig heavy variable 3-72 | *IGHV3-72* | 1.48 | 0.025 |  |  |  | Immune response |
| Immunoglobulin heavy constant δ | *IGHD* | 3.39 | 0.009 |  |  |  | Immune response |
| IGK@ protein | *IGK@* | 1.31 | 0.002 |  |  |  | Immune response |
| IgG-2A chain C region |  |  |  | *Ighg2c* | 0.32 | 9E-14 | Immune response |
| IgG-2B chain C region |  |  |  | *Ighg2b* | 0.43 | 1E-06 | Immune response |
| Ig heavy chain V region MOPC 21 |  |  |  | *Ighv5-17* | 0.40 | 5E-05 | Immune response |
| Ig-J chain | *IGJ* | 1.51 | 0.006 | *Igj* | 0.19 | 7E-12 | Immune response |
| Ig kappa chain C region |  |  |  | *Igkc* | 0.45 | 3E-08 | Immune response |
| Ig kappa chain V-III region PC 2880/PC 1229 |  |  |  | *Igkv3-2* | 0.17 | 1E-13 | Immune response |
| IgK variable | *IGKV2D-24* | 0.70 | 0.024 |  |  |  | Immune response |
| Ig kappa chain V-V region HP 93G7 |  |  |  | *N/A* | 0.25 | 2E-07 | Immune response |
| Ig kappa chain V-V region K2 |  |  |  | *N/A* | 0.33 | 3E-04 | Immune response |
| Ig kappa chain V-V region L6 |  |  |  | *N/A* | 0.22 | 1E-09 | Immune response |
| Ig kappa chain V-V region MOPC 149 |  |  |  | *N/A* | 0.22 | 4E-07 | Immune response |
| Ig kappa chain V-V region MOPC 41 |  |  |  | *Gm5571* | 0.09 | 2E-10 | Immune response |
| Ig lambda-1 chain C region |  |  |  | *N/A* | 0.58 | 6E-05 | Immune response |
| Ig lambda-2 chain C region |  |  |  | *Iglc2* | 0.44 | 1E-06 | Immune response |
| Ig mu chain C region |  |  |  | *Ighm* | 0.27 | 7E-18 | Immune response; complement activation (classical pathway) |
| Ig kappa variable 17-127 |  |  |  | *Igkv17-127* | 0.13 | 3E-06 | Immune response |
| Ig kappa variable 4-57-1 |  |  |  | *Igkv4-57-1* | 0.13 | 1E-05 | Immune response |
| Ig kappa variable 5-39 |  |  |  | *Igkv5-39* | 0.10 | 8E-14 | Immune response |
| Ig heavy variable 1-81 |  |  |  | *Ighv1-81* | 0.62 | 0.035 | Immune response |
| Ig kappa variable 16-104 |  |  |  | *Igkv16-104* | 0.40 | 0.001 | Immune response |
| Ig kappa variable 4-63 |  |  |  | *Igkv4-63* | 0.08 | 7E-08 | Immune response |
| Ig kappa variable 4-74 |  |  |  | *Igkv4-74* | 0.13 | 5E-08 | Immune response |
| Ig heavy variable V9-3 |  |  |  | *Ighv9-3* | 0.28 | 5E-06 | Immune response; complement activation (classical pathway) |
| Ig heavy variable 1-82 |  |  |  | *Ighv1-82* | 0.24 | 8E-08 | Immune response |
| Ig heavy variable 1-76 |  |  |  | *Ighv1-76* | 0.17 | 3E-08 | Immune response |
| Ig kappa chain variable 9-124 |  |  |  | *Igkv9-124* | 0.19 | 3E-05 | Immune response |
| Ig kappa chain variable 8-27 |  |  |  | *Igkv8-27* | 0.33 | 5E-07 | Immune response |
| Ig heavy variable 6-3 |  |  |  | *Ighv6-3* | 0.39 | 2E-05 | Immune response |
| Ig heavy variable 1-62-2 |  |  |  | *Ighv1-62-2* | 0.34 | 2E-04 | Immune response |
| Ig kappa chain variable 4-72 |  |  |  | *Igkv4-72* | 0.19 | 2E-06 | Immune response |
| Ig heavy variable 10-1 |  |  |  | *Ighv10-1* | 0.30 | 7.4E-08 | Immune response |
| Ig kappa variable 1-133 |  |  |  | *Igkv1-133* | 0.34 | 7E-05 | Immune response; complement activation (classical pathway) |
| Leucine-rich HEV glycoprotein |  |  |  | *Lrg1* | 1.77 | 2E-05 | Positive regulation of angiogenesis; Brown fat cell differentiation |
| Ig gamma-1 chain C region, membrane-bound |  |  |  | *Ighg1* | 0.69 | 0.010 | Immune response; complement activation |
| Membrane maltase-glucoamylase |  |  |  | *Mgam* | 0.37 | 2E-05 | Carbohydrate digestion/absorption |
| Complement factor H-related protein C |  |  |  | *Gm4788* | 0.52 | 3E-06 | Complement/coagulation cascades |
| Complement component 6 |  |  |  | *C6* | 1.99 | 0.033 | Complement activation, regulation /coagulation cascades |
| Insulin-like growth factor-binding protein complex acid labile subunit |  |  |  | *Igfals* | 0.77 | 0.004 | Cell adhesion |
| Inter-α-trypsin inhibitor heavy chain H2 | *ITIH2* | 0.92 | 0.022 | *Itih2* | 1.12 | 0.013 | Endopeptidase inhibitor |
| Inter-α-trypsin inhibitor heavy chain H3 |  |  |  | *Itih3* | 1.54 | 3E-07 | Endopeptidase inhibitor |
| Kininogen-1 | *KNG1* | 0.86 | 2E-04 |  |  |  | endopeptidase inhibitor; blood coagulation, intrinsic path |
| Leukemia inhibitory factor receptor |  |  |  | *Lifr* | 0.37 | 6E-08 | Cytokine-mediated signaling |
| Lumican |  |  |  | *Lum* | 1.39 | 2E-04 | Collagen binding, organization of cartilage development |
| Macrophage colony-stimulating factor 1 receptor |  |  |  | *Csf1r* | 1.51 | 0.012 | Cytokine-cytokine receptor interaction, Tyr protein kinase, innate immunity |
| Major urinary protein 20 |  |  |  | *Mup20* | 5.75 | 6E-06 | Associative learning; Behavior |
| Mannose-binding protein A |  |  |  | *Mbl1* | 0.64 | 0.046 | Complement/coagulation cascades; Phagosome |
| Mannose-binding protein C |  |  |  | *Mbl2* | 1.23 | 0.014 | Complement activation (classical & lectin pathways)/coagulation cascades; Phagosome |
| Peptidase inhibitor 16 |  |  |  | *Pi16* | 2.05 | 8E-05 | Negative regulation of cardiac muscle growth |
| Phosphatidylcholine-sterol acyltransferase |  |  |  | *Lcat* | 1.90 | 1E-11 | Plasma lipoprotein metabolism |
| Phosphatidylinositol-glycan-specific phospholipase D |  |  |  | *Gpld1* | 1.26 | 2E-04 | Glycosylphosphatidylinositol-anchor biosynthesis |
| Phospholipid transfer protein |  |  |  | *Pltp* | 2.05 | 7E-04 | Lipid transport |
| Plasma kallikrein | *KLKB1* | 0.91 | 0.043 | *Klkb1* | 0.73 | 8E-07 | Complement/coagulation cascades |
| Plasma protease C1 inhibitor |  |  |  | *Serping1* | 1.63 | 2E-10 | Complement/coagulation cascades |
| Protein AMBP |  |  |  | *Ambp* | 1.26 | 0.002 | Protein catabolic process; Inhibits trypsin, plasmin, elastase |
| Protein Z-dependent protease inhib |  |  |  | *Serpina10* | 1.67 | 2E-07 | Blood coagulation, neg. regulator |
| Prothrombin | *F2* | 0.79 | 9E-06 |  |  |  | Complement/coagulation cascades |
| Pyrethroid hydrolase Ces2e |  |  |  | *Ces2e* | 7.57 | 9E-10 | Response to stimulus; carboxylic esterase |
| Selenoprotein P |  |  |  | *Sepp1* | 1.45 | 2E-07 | Brain development; sexual reproduction |
| Serine protease inhibitor A3N |  |  |  | *Serpina3n* | 1.70 | 4E-11 | Acute phase response |
| Serotransferrin |  |  |  | *Tf* | 1.25 | 4E-07 | Iron ion homeostasis; Actin filament organization |
| Serum amyloid P-component |  |  |  | *Apcs* | 5.89 | 7E-09 | Immune response |
| Serum amyloid A-1 | *SAA1* | 1.97 | 0.020 |  |  |  | Acute phase response |
| Serum paraoxonase 1 |  |  |  | *Pon1* | 0.59 | 0.002 | Homocysteine thiolactone detoxification |
| Sulfated glycoprotein 1, Prosaposin |  |  |  | *Psap* | 2.47 | 9E-09 | Lysosome |
| Sulfhydryl oxidase 1 |  |  |  | *Qsox1* | 1.37 | 2E-07 | Cell redox homeostasis |
| Thrombospondin-1 |  |  |  | *Thbs1* | 2.31 | 0.006 | MAPK activation; ER steress resp.; Cell adhesion; Fbg binding; downregulation of fibrinolysis |
| Thyroxine-binding globulin |  |  |  | *Serpina7* | 6.17 | 2E-11 | Thyroid hormone transport; Aging |
| Transthyretin | *TTR* | 0.57 | 5E-09 |  |  |  | Retinol metabolic process, thyroid hormone transport |
| Vitamin D-binding protein | *GC* | 0.85 | 1E-04 | *Gc* | 1.43 | 2E-13 | Vitamin metabolic process |
| Vitamin K-dependent protein S |  |  |  | *Pros1* | 1.31 | 0.031 | Blood coagulation; Fibrinolysis |
| Vitronectin |  |  |  | *Vtn* | 1.47 | 1E-11 | Cell migration; Cell adhesion; Immune response |
| Zinc-alpha-2-glycoprotein |  |  |  | *Azgp1* | 1.36 | 5E-10 | Cell adhesion |
| * Data for human *CBS*^-/-^ patients and controls are from Sikora *et al*., 2019.  Text highlighted in yellow indicates proteins detected also as non-differentiating in mice.  Text highlighted in green indicates proteins detected also as non-differentiating in human. | | | | | | | |
